# Biochemical quantitation of the eIF5A hypusination in *Arabidopsis thaliana* uncovers ABA-dependent regulation

**DOI:** 10.3389/fpls.2014.00202

**Published:** 2014-05-16

**Authors:** Borja Belda-Palazón, María A. Nohales, José L. Rambla, José L. Aceña, Oscar Delgado, Santos Fustero, M. Carmen Martínez, Antonio Granell, Juan Carbonell, Alejandro Ferrando

**Affiliations:** ^1^Instituto de Biología Molecular y Celular de Plantas, CSIC-Universidad Politécnica de ValenciaValencia, Spain; ^2^Centro de Investigación Príncipe FelipeValencia, Spain; ^3^Departamento de Química Orgánica, Universidad de ValenciaValencia, Spain; ^4^Departament de Bioquímica i Biologia Molecular, Universitat Autònoma de BarcelonaBarcelona, Spain

**Keywords:** spermidine, hypusine, eIF5A, 2D-electrophoresis, abscisic acid

## Abstract

The eukaryotic translation elongation factor eIF5A is the only protein known to contain the unusual amino acid hypusine which is essential for its biological activity. This post-translational modification is achieved by the sequential action of the enzymes deoxyhypusine synthase (DHS) and deoxyhypusine hydroxylase (DOHH). The crucial molecular function of eIF5A during translation has been recently elucidated in yeast and it is expected to be fully conserved in every eukaryotic cell, however the functional description of this pathway in plants is still sparse. The genetic approaches with transgenic plants for either eIF5A overexpression or antisense have revealed some activities related to the control of cell death processes but the molecular details remain to be characterized. One important aspect of fully understanding this pathway is the biochemical description of the hypusine modification system. Here we have used recombinant eIF5A proteins either modified by hypusination or non-modified to establish a bi-dimensional electrophoresis (2D-E) profile for the three eIF5A protein isoforms and their hypusinated or unmodified proteoforms present in *Arabidopsis thaliana*. The combined use of the recombinant 2D-E profile together with 2D-E/western blot analysis from whole plant extracts has provided a quantitative approach to measure the hypusination status of eIF5A. We have used this information to demonstrate that treatment with the hormone abscisic acid produces an alteration of the hypusine modification system in *Arabidopsis thaliana*. Overall this study presents the first biochemical description of the post-translational modification of eIF5A by hypusination which will be functionally relevant for future studies related to the characterization of this pathway in *Arabidopsis thaliana*.

## Introduction

The eukaryotic cell contains polyamines, metabolites derived from amino acid catabolism that behave as polycations at physiological pH. Among the most abundant natural polyamines, the triamine spermidine has been shown to be essential for cell viability (Hamasaki-Katagiri et al., 1997; Imai et al., 2004; Mandal et al., 2013). Genetic alterations of spermidine metabolism in *S. cerevisiae* have shown that one crucial function for spermidine to support growth is the modification of the translation elongation factor eIF5A (Chattopadhyay et al., 2008). In fact, the biological activity of eIF5A is dependent on spermidine by means of a well-characterized post-translational enzymatic modification named hypusination with the sequential intervention of two enzymes namely deoxyhypusine synthase (DHS) and deoxyhypusine hydroxylase (DOHH) (Park, 2006; Wolff et al., 2007). In the limiting first reaction, the enzyme DHS catalyzes the NAD-dependent cleavage and transfer of the aminobutyl moiety of the spermidine to the ε-amino group of one conserved lysine of eIF5A to form an intermediate residue named deoxyhypusine. In the second reaction that intermediate is hydroxylated by the Fe(II)-dependent enzyme DOHH to yield the hypusine residue in the active and mature eIF5A proteoform. The activity of eIF5A itself is essential for cell survival in eukaryotes (Kang and Hershey, 1994; Park et al., 1997; Nishimura et al., 2002, 2012; Pagnussat et al., 2005; Feng et al., 2007). eIF5A has been postulated as an RNA-binding protein involved in mRNA transport and metabolism (Xu and Chen, 2001; Xu et al., 2004; Li et al., 2010; Maier et al., 2010). However, the best characterized cellular activity for eIF5A is its function as a translation factor involved in the elongation step (Gregio et al., 2009; Saini et al., 2009). Recent studies have elucidated a more detailed function within the ribosome for eIF5A and EF-P, a prokaryotic structural homolog. These proteins are required in their respective systems for the translation of mRNAs encoding clusters of consecutive proline residues that cause ribosome stalling (Doerfel et al., 2013; Gutierrez et al., 2013). The characterization of the eIF5A pathway in plants has focused on genetic approaches to overexpress or knock-down either the eIF5A genes or the modifying enzyme DHS. These studies have proposed functions for eIF5A related to either developmental or stress-induced cell death processes mostly characterized in *A. thaliana* whose genome carries three genes encoding very similar eIF5A proteins (Duguay et al., 2007; Feng et al., 2007; Liu et al., 2008; Ma et al., 2010; Xu et al., 2011; Wang et al., 2012; Ren et al., 2013). However, despite the availability of detailed functional genetic data, there is a lack of molecular evidence for eIF5A activity in plants. This may be in part due to the absence of biochemical tools to evaluate the eIF5A activity since the identity of the mRNAs regulated at the post-transcriptional level by eIF5A are unknown in plants.

One approach to understand eIF5A activity relies in its complex post-translational modifications as it has been reported that eIF5A can be subjected to phosphorylation, acetylation, ubiquitylation, and hypusination that regulate its stability, subcellular localization, and functional activity (Park et al., 1993; Jin et al., 2003; Lee et al., 2009; Łebska et al., 2010; Ishfaq et al., 2012). The hypusination of eIF5A yields a modified lysine residue with increased molecular weight and altered isoelectric point that can be used to biochemically distinguish both proteoforms (Klier et al., 1995). In this work we have generated recombinant versions of hypusinated and non-hypusinated eIF5A proteins from *Arabidopsis thaliana* that have been used to establish a biochemical profile of the different eIF5A proteins and their proteoforms by means of 2D-E and western blot analysis. We have also applied this biochemical technique to show that the plant stress hormone abscisic acid causes a reduction in the hypusination of eIF5A1 probably through the post-transcriptional alteration of DHS activity.

## Materials and methods

### Plant material and growth conditions

*Arabidopsis* wild type (Col-0) and *CKA3*^*mut*^ plants were grown *in vitro* with solid MS medium containing 2.45 g/L MS salts (Duchefa, The Netherlands) and 6 mM MES buffer adjusting pH 5.7 with KOH and solidified with 1% Phyto Agar. When needed, ABA plant hormone (Sigma, USA) or dexamethasone (Sigma, USA) was added as indicated. *Arabidopsis* seeds were surface sterilized by mixing around 100 seeds with 300 μL of 70% ethanol and 0.05% Triton X-100 for 5 min while shaking. After centrifugation and supernatant removal, the seeds were incubated for 5 min shaking with the same volume of 96% ethanol and sown by pipetting on sterile filter paper under laminar flow for 15 min for ethanol evaporation. Once dried the seeds were sawn on MS medium in Petri dishes sealed with Micropore (3M, USA) and stratified for 3 days at 4°C before being cultivated in growth chamber under long day photoperiod conditions (16 h light intensity 110 μmol m^−2^ s^−1^ and 8 h dark) at 22°C ± 1°C.

### Cloning procedures

The cloning procedures for heterologous expression in *E. coli* as translational fusions to either His or GST-tags were carried out following a two-step sequential PCR as previously described (Belda-Palazón et al., 2012). Gene coding sequences of eIF5A1, eIF5A2, eIF5A3, DHS, and DOHH were PCR-amplified from *A. thaliana* cDNA using the following primers for eIF5A1: 5′- GG ACA AGT TTG TAC AAA AAA GCA GGC TTA ATG TCC GAC GAG GAG CAT CAC− 3′ and 5′- GG AC CAC TTT GTA CAA GAA AGC TGG GTC CTT GGG ACC GAT GTC CTT AAG− 3′; for eIF5A2: 5′- GG ACA AGT TTG TAC AAA AAA GCA GGC TTA ATG TCT GAC GAC GAG CAC CAC− 3′ and 5′- GG AC CAC TTT GTA CAA GAA AGC TGG GTC CTT GCC ACC ACC AAC TTC CTT− 3′; for eIF5A3: 5′- GG ACA AGT TTG TAC AAA AAA GCA GGC TTA ATG TCA GAC GAC GAG CAT CAC− 3′ and 5′- GG AC CAC TTT GTA CAA GAA AGC TGG GTC CTT GGG ACC AAC TTC CTT GAG− 3′; for DHS: 5′- GG ACA AGT TTG TAC AAA AAA GCA GGC TTA ATG GAG GAT GAT CGT GTT TTC− 3′ and 5′- GG AC CAC TTT GTA CAA GAA AGC TGG GTC AGT CTT AGA CTC ACA GGT TTG− 3′; for DOHH: 5′- GG ACA AGT TTG TAC AAA AAA GCA GGC TTA ATG GAA TCT AAT GGA TCA GTT− 3′ and 5′- GG AC CAC TTT GTA CAA GAA AGC TGG GTC GTG AAC AAG CGG GTC TTG CGT− 3′ and the PCR products were used to obtain the entry clones in pDONR-Zeo (Invitrogen, USA). The entry clones were subcloned into pDEST15 (Invitrogen, USA) for heterologous GST fusion protein expression without post-translational modifications. To obtain the recombinant eIF5A hypusinated proteins, the entry clones containing *eIF5A* genes were subcloned into pQlinkGD (Addgene plasmid #13673) to obtain GST fusion proteins, whereas both entry clones with the modification enzymes DHS and DOHH were subcloned into pQlinkHD (Addgene plasmid #13668) to yield His-tag enzymes. pQlink vectors provided by Addgene were a gift of Kondrad Buessow and all ligation-independent cloning methods were followed as reported (Scheich et al., 2007). In a first step DOHH-His was subcloned as a *Pac*I restriction fragment into *Swa*I-digested pQlinkHD-DHS to obtain His-DHS/His-DOHH double construct. Subsequently this double construct linearized with *Swa*I was used for the subcloning of *Pac*I restriction fragments of each GST-eIF5A construct to yield the three final constructs DHS/DOHH/eIF5A1-pQlink, DHS/DOHH/eIF5A2-pQlink, and DHS/DOHH/eIF5A3-pQlink. Each final construct was introduced into *E. coli* strain BL21-CodonPlus (DE3) (Stratagene-Agilent, USA) for recombinant heterologous protein expression. Nucleic acid handling and plasmid images were performed with software VectorNTI Suite 9 (Invitrogen, USA).

### Recombinant protein purification and plant protein extraction

Bacterial growth conditions and isopropyl-β-D-thiogalactopyranoside (IPTG) induction were performed as described (Belda-Palazón et al., 2012). Collected cells were resuspended with 0.5 mL extraction buffer containing 20 mM Tris-HCl pH 7.6, 150 mM NaCl, 1 mM DTT and protease inhibitor cocktail (Sigma, USA), sonicated, and centrifuged for 15 min to remove cell debris and insoluble material. To purify GST-tag proteins, total soluble proteins were loaded on a 1 mL Glutathione agarose resin (ABT, Spain) and incubated overnight at 4°C. After exhaustive washing, the resin-bound GST-tag proteins were eluted twice by incubation at 4°C for 5 min with 1 mL elution buffer containing 50 mM Tris-HCl pH 8.0 and 10 mM L-glutathione. To remove excess glutathione and concentrate the protein, the samples were washed with TBS buffer (20 mM Tris-HCl and 150 mM NaCl) through Vivaspin 5 kDa MWCO (GE Healthcare, USA) according to the manufacturer instructions. To cleave the GST tag, the GST-eIF5A purified proteins were bound again to the Glutathione agarose beads and, after washing with 20 mM Tris-HCl, 150 mM NaCl, 1 mM DTT, and 1 mM EDTA, cleavage of the GST was achieved by overnight treatment at 4°C with Protease 3C PreScission protease (GE Healthcare, USA) according to the manufacturer. After collecting the soluble released eIF5A proteins, the remaining bound GST and Protease 3C were eluted by incubation with elution buffer.

For plant protein extraction, plant seedlings were ground in lysis buffer containing 50 mM Tris-HCl pH 7.6, 10% glycerol, 1 mM DTT, 1 mM EDTA, and 0.5% Igepal containing plant protease inhibitor cocktail (Sigma, USA). The extract was centrifuged at least twice at 3000 g for 10 min at 4°C to remove cell debris and if needed filtered through 0.45 μm polypropylene filter (VWR, USA). Total soluble proteins were precipitated by mixing for 1 h at 4°C with an equal amount of cold 20% TCA (trichloroacetic acid, Sigma). After centrifugation at 10,000 g for 15 min, pellets were washed three times with cold acetone and finally resuspended by vortex and sonication in 2D-lysis buffer containing 7 M urea, 2 M thiourea, and 4% CHAPS. Non-protein contaminants were removed using the 2D Clean-Up Kit according to the manufacturer (GE Healthcare, USA) and resolubilized in the 2D-lysis buffer for determination of protein concentration with the Bio-Rad (USA) protein assay.

### Protein fluorescent labeling and 2D electrophoresis

Recombinant hypusinated and non-hypusinated eIF5A proteins were labeled using the CyDyes DIGE fluors (Cy2, Cy3, and Cy5) according to the manufacturer (GE Healthcare, USA). Equal amounts (1 μg) of each fluorescently labeled protein were pooled and 2D-lysis buffer was added to a final volume of 40 μL. The samples were then mixed with 40 μL of isoelectrofocusing (IEF) rehydration buffer (8 M urea, 4% CHAPS, 0.005% bromophenol blue) containing 65 mM DTT and 1% IPG buffer pH 3–11. For 2D-E analysis, strips of 7 cm in length with immobilized 4–7 pH gradient were hydrated overnight at room temperature with 125 μL of IEF buffer containing the reagents Destreak and Pharmalyte according to the manufacturer (GE Healthcare, USA). Protein samples (15 μg of total plant protein or 1 μg fluorescently labeled recombinant protein) were loaded in hydrated strips. IEF was performed on an IPGphor unit (GE Healthcare, USA) at 20°C and a maximum current setting of 75 μA per strip, using the following settings: 300 V for 45 min, an increasing voltage gradient to 1000 V over 25 min, an increasing voltage gradient to 5000 V over 2 h, before finally holding at 5000 V for a total of 7000 Vh. After IEF, each strip was equilibrated separately for 15 min in 10 mL equilibration solution I (0.05 M Tris-HCl buffer, pH 8.8 containing 6 M urea, 30% glycerol, 2% SDS, 200 mg DTT per 10 mL buffer) followed by equilibration solution II (substituting DTT for 250 mg iodoacetamide per 10 mL buffer and adding 0.01% bromophenol blue) before being applied directly to the second dimension 14% SDS-PAGE gels. After SDS-PAGE, gels were either coomassie-stained or used for immunoblot analysis. CyDye-labeled proteins were visualized by scanning using a Typhoon Trio scanner (GE Healthcare, USA) with the relevant wavelengths for each CyDye. Cy2 image were scanned using a blue laser (488 nm) and an emission filter of 520 nm band pass (BP) 40. Cy3 image were scanned using a green laser (532 nm) and a 580 nm BP 30 emission filter. Cy5 image were scanned using a red laser (633 nm) and a 670 nm BP 30 emission filter. All gels were scanned at 200 μm (pixel size) resolution. The photomultiplier tube (PMT) was set between 500 and 600 V by using normal sensitivity. The scanned gels were directly transferred to the ImageQuant V5.2 software package (GE Healthcare, USA).

### Antibody production, immunological methods, and quantitative RT-PCR

To raise polyclonal antibodies against eIF5A1 protein, 2.5 mg of the non-modified recombinant purified protein without GST tag were sent to BioGenes GmbH (Germany). The polyclonal antiserum produced by the company was tested at different dilutions in western blots to be optimally used at 1:5000. The same 1:5000 working dilution was used with secondary anti-rabbit antibody coupled to peroxidase (Santa Cruz Biotechnology, USA). Immunoblot detection was done with the chemiluminescent ECL detection kit (GE Healthcare, USA). For quantitative gene expression studies based on reverse transcriptase and quantitative PCR (RTqPCR) were performed as described (Cuevas et al., 2008). Primers used for qPCR are shown in Table 1 for the genes *DHS*, *eIF5A1*, *eIF5A2*, and *eIF5A3*. Primers for the reference gene *PDF2* and the ABA-inducible genes *RAB18* and *RD29B* were previously published (Czechowski et al., 2005; Saez et al., 2006; Cuevas et al., 2008).

**Table 1:** **Primers for real-time qPCR**.

**Primer**	**Sequence**
DHS-F	CTCACAGGTTTGGTCTCTCTTTG
DHS-R	GTTCTGCTAAAACCGTTAAGGTATAC
eIF5A1-F	GGAGAGGAACAGATCAATGCTC
eIF5A1-R	GAGTAATGGAAGCCTACAGAAGAAG
eIF5A2-F	TGCTCACTTCTCTCTCTTAGG
eIF5A2-R	GATTCGCTGGCCTCAAAG
eIF5A3-F	AGGATATTGTTGTGTCTGTCATG
eIF5A3-R	TTATTATTATTACTTGGGACCAACTTC

### MALDI-TOF and GC-MS

1.5 μg of recombinant hypusinated and non-hypusinated eIF5A proteins were digested with sequencing grade trypsin (Promega, USA) as described (Shevchenko et al., 1996). For the MALDI TOFTOF analysis, 1 μL of the digestion mixture was spotted onto the MALDI target plate. After the droplets were air-dried at room temperature, 1 μL of matrix containing 5 mg/mL CHCA (α-cyano-4-hydroxycinnamic acid, Bruker, USA) in 0.1% TFA (trifluoroacetic acid)-ACN (acetonitrile)/H_2_O (1:1 v/v) was added and allowed to air-dry at room temperature. The resulting mixtures were analyzed in a 5800 MALDI TOFTOF (ABSciex, USA) in positive reflection mode (3000 shots every position). Previously the plate and the acquisition methods were calibrated with 0.5 μL of the calibration mixture (ABSciex, USA) in 13 positions. The MS information was sent to MASCOT via the Protein Pilot (ABSciex, USA) and was analyzed manually with mMass v5.2.0 (Strohalm et al., 2008). The MALDI TOFTOF analysis was carried out in the SCSIE Proteomics laboratory of the Universidad de Valencia, a member of ISCIII Carlos III Network Proteomics Platform. The mass spectrometry proteomics data have been deposited to the ProteomeXchange Consortium (http://www.proteomexchange.org) via the PRIDE partner repository (Vizcaíno et al., 2013) with the dataset identifier PXD000880 and DOI 10.6019/PXD000880.

For the GC-MS studies, 0.4 mg of purified recombinant GST-eIF5A2 protein, hypusinated or non-hypusinated, were eluted in TBS buffer. 1 volume of 37% HCl was added and the solution was flame sealed in a glass vial, agitated, and incubated at 110°C for 24 h for complete acid hydrolysis. The resulting amino acid solution was dried in a speed-vacuum overnight, and derivatized by trimethylsilylation with MSTFA in pyridine as described (Zanor et al., 2009). Two microliters of the trimethylsilylated hydrolyzed protein were injected in splitless mode in a 6890 N gas chromatograph fitted with a secondary oven and a cryogenic modulator (Agilent Technologies, USA) coupled to a Pegasus 4D TOF mass spectrometer (LECO, USA). The 2D separation was achieved using a RTX-5 (10 m × 0.18 mm × 0.20 μm) (Restek, USA) 1st-dimension column in the primary oven and a DB-17 (1.1 m × 0.10 mm × 0.10 μm) 2nd-dimension column in the secondary oven. The liner was set at 270°C. Helium was used as carrier gas with a constant flow of 1 mL min^−1^. Primary oven program was 70°C for 2 min, 20°C min^−1^ ramp until 260°C, and 260°C for 5 min. Secondary oven temperature was programmed to maintain a temperature 20°C higher than the primary oven during all the run. Mass spectra were collected at 100 scans s^−1^ in the m/z range 60–800. Ion source was set at 200°C and ionization energy at 70 eV. Identification of trimethylsilylated hypusine, deoxyhypusine, and other amino acids was based on comparison of both mass spectrum and retention index with those of pure standards. Hypusine and deoxyhypusine standards were chemically synthesized according to previously reported procedures (Bergeron et al., 1998).

## Results

### Recombinant expression and biochemical separation of hypusinated and non-hypusinated eIF5A proteins from *A. thaliana*

**Figure 1:**
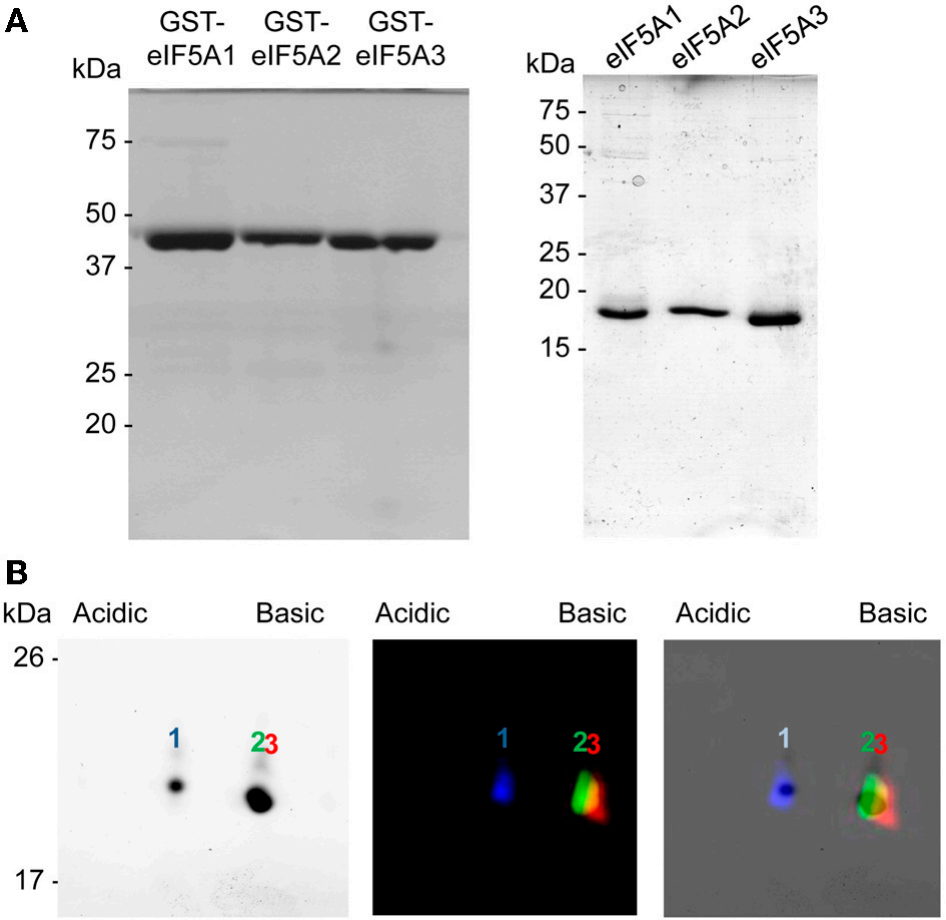
**Recombinant protein expression and 2D-E separation of non-modified eIF5A proteins. (A)** GST-fusion proteins (left) or GST-cleaved eIF5A (right) recombinant proteins were separated by 1D SDS-PAGE and coomasie stained. **(B)** Recombinant eIF5A proteins were labeled with fluorescent dyes eIF5A1-Cy2, eIF5A2-Cy3, and eIF5A3-Cy5, mixed and separated by 2D-E for coomassie staining (left) or fluorescent scanning (middle), and both images were merged (right).

To evaluate differences in the 2D-E mobility of the three *A. thaliana* eIF5A proteins we cloned and expressed the corresponding cDNAs in *E. coli*, which does not contain any homologous proteins nor the hypusination enzymes DHS and deoxyhpusine hydroxylase (DOHH). The three cDNAs encoding eIF5A proteins were cloned and expressed as gluthathione-S-transferase (GST) fusion proteins including the Protease 3C-Prescission target sequence for cleavage of the GST tag that can be used to release the soluble eIF5A full length proteins (Figure 1A). All three recombinant proteins eIF5A1, eIF5A2, and eIF5A3 were labeled as full length proteins with different fluorescent dyes to follow their position after 2D-E. After mixing all three recombinant labeled proteins they were subjected to 2D-E and either coomassie staining or fluorescent scanning to distinguish the relative mobility of each protein. As shown in Figure 1B, and as expected from the theoretical isoelectric points, the eIF5A1 protein (pI 5.532) is more acidic than the eIF5A2 (pI 5.813) and eIF5A3 (pI 5.816) proteins that display a very close pI with a slightly less acidic value for the eIF5A3 protein. These data indicate that the 2D-E technique can effectively separate eIF5A1 protein from eIF5A2 and eIF5A3 at least in their non-hypusinated proteoforms.

**Figure 2:**
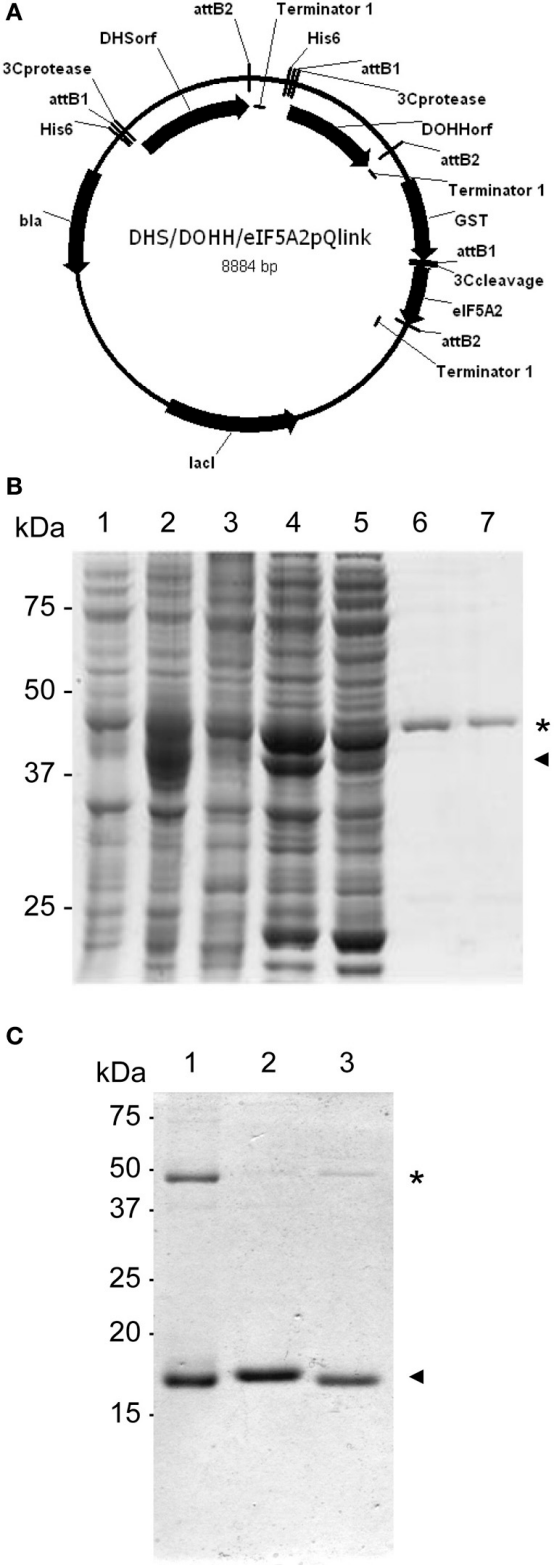
**Coexpression of GST-eIF5A proteins and hypusination enzymes. (A)** Schematic diagram of the resulting plasmid after LIC cloning into pQlink of *GST-eIF5A2* together with *His-DHS* and *His-DOHH*. **(B)** SDS-PAGE analysis of protein fractions for purification of hypusinated GST-eIF5A2. The coomassie staining SDS-PAGE contains in lane 1 a total non-induced protein extract, lane 2 IPTG-induced total protein extract, lane 3 non-induced total soluble protein extract, lane 4 IPTG-induced soluble protein extract, lane 5 unbound protein extract to glutathione sepharose, lanes 6 and 7 soluble protein extract after elution by glutathione competition. Asterisk indicates the presence of GST-eIF5A2 (45 kDa) partially overlapping with His-DHS (44 kDa), and arrowhead corresponds to His-DOHH (37 kDa). **(C)** Partially hypusinated GST-eIF5A proteins immobilized on glutathione sepharose were cleaved with Protease3C and the resulting soluble fractions were separated on SDS-PAGE and coomassie stained to show the purification of eIF5A1 (lane 1), eIF5A2 (lane 2) and eIF5A3 (lane 3) as indicated with arrowheads. Residual GST-eIF5A proteins are still present in the preparations as indicated with asterisks.

**Figure 3:**
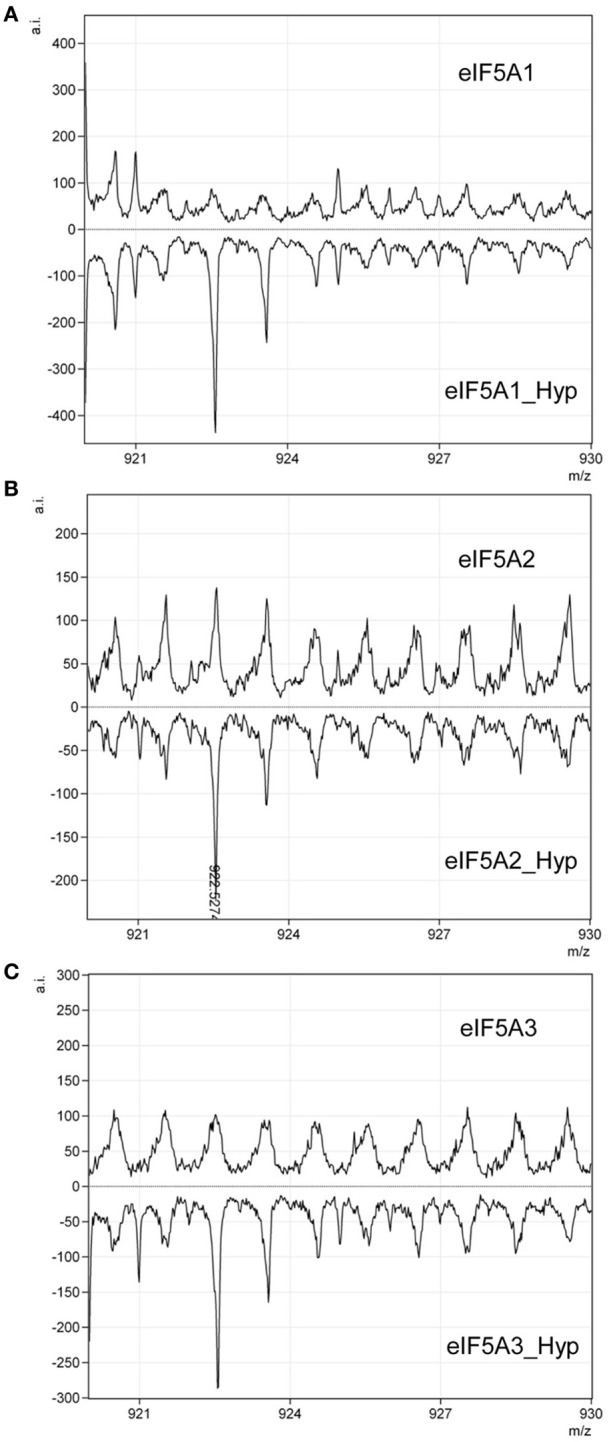
**Qualitative assessment of eIF5A hypusination by MALDI-TOF**. Recombinant eIF5A proteins modified by hypusination or unmodified: eIF5A1 **(A)**, eIF5A2 **(B)**, and eIF5A3 **(C)**, were subjected to trypsin digestion and checked by MALDI-TOF for the presence or absence of a tryptic peptide of m/z 922.5. Only hypusine-modified proteins showed the presence of the peptide of the expected m/z as indicated in **(B)**.

One remaining question was related to the apparent 2D-E mobility of the hypusinated proteins. The successful production of active recombinant eIF5A in *E. coli* by coexpression with the modification enzymes has been reported (Park et al., 2011), accordingly we pursued the simultaneous coexpression of eIF5A proteins with the hypusination enzymes DHS and DOHH by cloning the corresponding cDNAs into the pQLink vectors that make use of the ligation-independent cloning technique (Scheich et al., 2007). Figure 2A shows a schematic picture of the multiple cloning construct and Figure 2B displays the purification of recombinant GST-eIF5A2 co-expressed together with the tagged hypusination enzymes His-DHS and His-DOHH. Similar experiments were done for GST-eIF5A1 and GST-eIF5A3 and finally all three GST-fusion proteins were cleaved by treatment with Protease 3C to release the soluble eIF5A proteins as shown in Figure 2C. To evaluate whether the eIF5A proteins were indeed hypusinated, two different approaches were followed. First we took advantage of the total conservation of the primary sequence of the tryptic peptide that includes the hypusinated K among distinct eukaryotic cells (TGK^Hyp^HGHAK) that has been previously shown to resist trypsin cleavage when the otherwise susceptible K is modified by hypusination (Jin et al., 2003). We anticipated the presence of a tryptic peptide of m/z 922.5 if hypusination had occurred in the recombinant expressed eIF5A proteins. Both eIF5A proteins coexpressed with enzymes or not, were subjected to trypsin digestion and subsequent MALDI-TOF-MS analysis for the identification of the hypusinated tryptic peptide. As shown in Figure 3, one peptide of m/z 922.5 was identified only in the protein samples from the eIF5A proteins coexpressed with the hypusination enzymes and absent in the analysis when they were not. To further confirm that hypusine was present in those eIF5A proteins, an additional amino acid analysis was carried out with coexpressed GST-eIF5A3 fusion protein vs. GST-eIF5A3 that was not coexpressed. The analysis was performed by means of total amino acid hydrolysis and GCxGC-TOF-MS using chemically synthesized hypusine as a standard. As it is shown in Figure S1 only the coexpressed GST-eIF5A3 recombinant protein showed the presence of hypusine, thus confirming that the recombinant coexpressed proteins do contain the amino acid hypusine.

**Figure 4:**
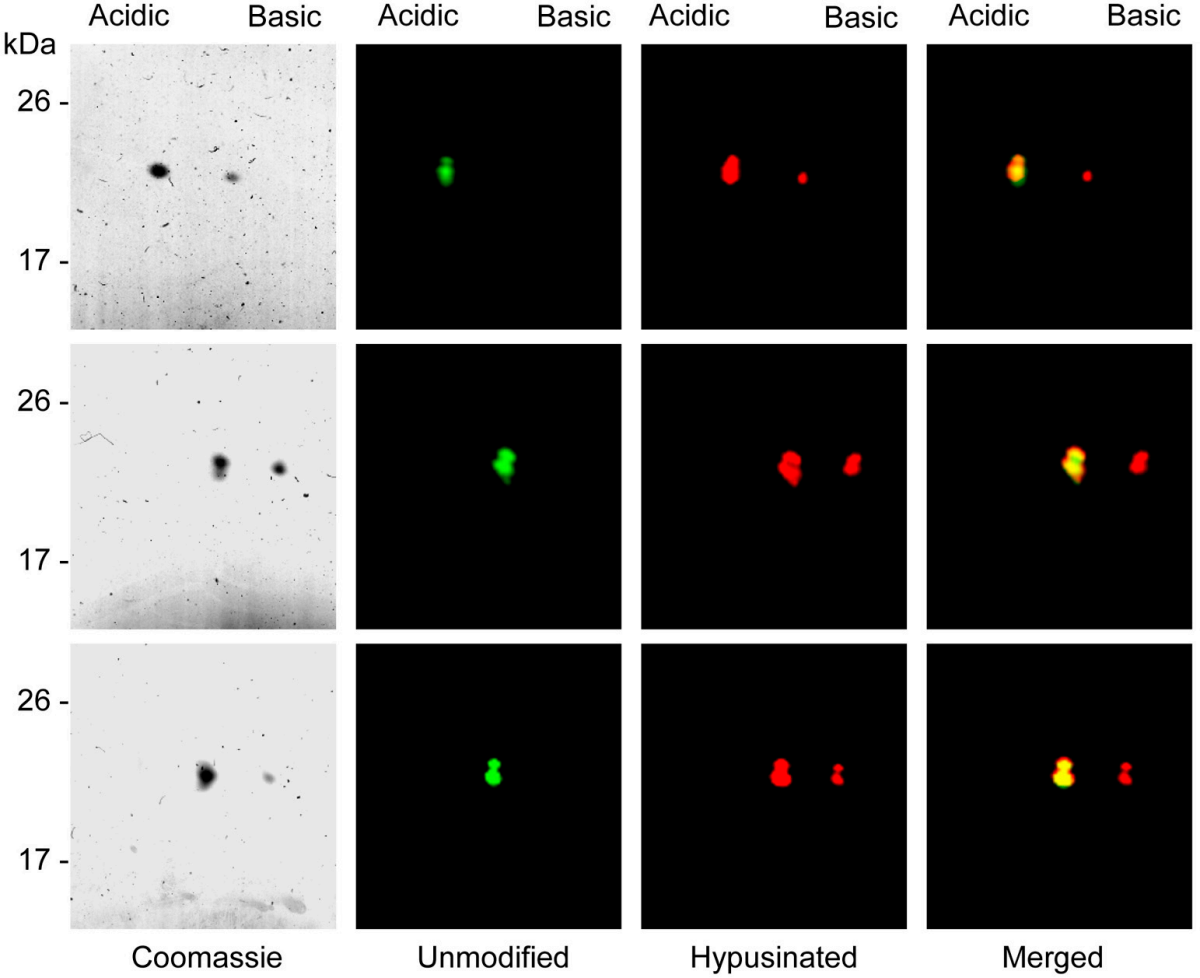
**Separation of unmodified and hypusine-modified eIF5A proteins by 2D-E**. eIF5A unmodified proteins were labeled with Cy3 dye and partially hypusine-modified eIF5A proteins were labeled with Cy5 fluorescent dye, mixed and separated by 2D-E. After fluorescent scanning the panel with unmodified proteins (green) shows a unique spot, whereas the panel with partially hypusine-modified proteins (red) shows two discrete spots. The merging of both fluorescent scannings shows the co-migration of the unmodified proteins with the most acidid spot of the hypusinated proteins. **Upper panels** correspond to eIF5A1, **central panels** to eIF5A2, and **lower panels** to eIF5A3 recombinant proteins.

Once the presence of hypusine in the eIF5A proteins coexpressed with enzymes was confirmed, a differential fluorescent labeling was done for each of the three eIF5A proteins coexpressed or not, and subjected to 2D-E and either coomassie stained or fluorescent scanning. The results, shown in Figure 4 showed that all three coexpressed eIF5A proteins displayed two spots with different pI. When compared with the mobility of the non-coexpressed recombinant proteins, a perfect overlap was found between the unmodified eIF5A and the acidic spot of the hypusinated protein, thus suggesting that the less acidic spot should correspond to the hypusinated version of the eIF5A protein. This was confirmed in a second MALDI-TOF-MS test by picking and trypsin-digestion of the coomassie stained spots (more acidic and less acidic) from the hypusine-modified eIF5A2 gel showing identical results as those shown in Figure 3B. These 2D-E data also confirmed that the hypusinated eIF5A proteins were not completely modified, since a relevant percentage of the protein corresponded to the more acidic unmodified spot. This is the case for all three eIF5A proteins with variable amounts of hypusination ranging between 20 and 45%. We tested alternative protocols for bacterial growth conditions changing both the media content and temperature, but no relevant improvements on the percentage of hypusination were achieved (data not shown). In spite of the partial modification of the recombinant eIF5A proteins, these proteins could be used to place the position of each eIF5A protein in their unmodified (more acidic spot) or hypusine-modified (less acidic spot) versions from a total protein extract from *A. thaliana* subjected to 2D-E analysis.

### Characterization of the proteomic profile of *A. thaliana* eIF5A by 2D-E and western blot

**Figure 5:**
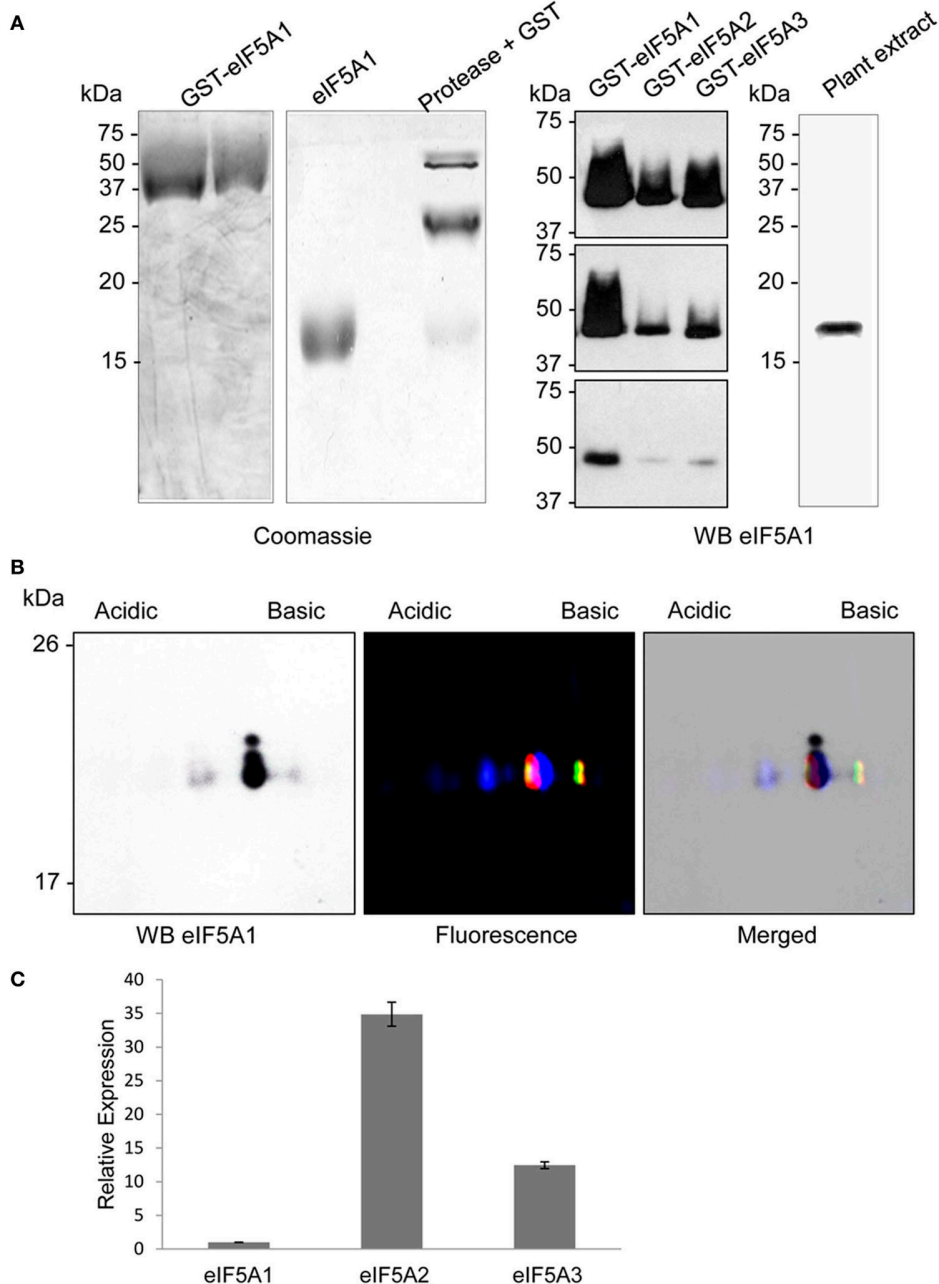
**Antibody production and immunological detection of eIF5A proteins**. Recombinant unmodified GST-eIF5A1protein was purified by glutathione sepharose affinity chromatography and cleaved with Protease 3C to yield highly pure eIF5A1 protein (coomassie stained gels on **A**). The polyclonal antibody generated against eIF5A1 protein was used to check cross-reactivity to recombinant GST-eIF5A proteins (1 μg, 500 ng, and 50 ng in the upper, medium, and lower panel, respectively) and immunological detection on total plant protein extract by western blot. Panel **(B)** shows the comparison of western blot detection of *Arabidopsis* total protein extract after 2D-E (left) with the fluorescent scanning (middle) of the 2D-E separation of fluorescent labeled recombinant hypusine-modified eIF5A proteins (eIF5A1 labeled with Cy2, eIF5A2 labeled with Cy3, and eIF5A3 labeled with Cy5), and both merged images (right). Panel **(C)** shows RTqPCR gene expression analysis for eIF5A1, eIF5A2, and eIF5A3 genes relative to eIF5A1 gene expression for *A. thaliana* plant seedlings.

To develop a 2D-E based test from a plant protein extract, we needed first to raise antibodies against the eIF5A proteins. With this purpose we performed a large scale purification of recombinant eIF5A1 protein as shown in Figure 5A. The GST-eIF5A1 purified fusion protein was cleaved with Protease 3C to yield highly pure eIF5A1 protein that was used to raise polyclonal antibodies. In a western blot analysis against recombinant unmodified eIF5A proteins, the antibody was shown to recognize optimally the eIF5A1 protein but also to partially cross-react against eIF5A3 and to a minor extent also with eIF5A2 (Figure 5A). When testing by western blot diluted amounts of the recombinant proteins, differences in the detection limit were observed. Figure 5A shows an enhanced detection level for eIF5A1 compared to eIF5A2 or eIF5A3 proteins whose signals are neglectable in the low nanogram range. In a western blot analysis with total protein extract from *A. thaliana* seedlings, a single band of the expected size for eIF5A1 was immunodecorated showing the specificity of the antibody in a plant total protein extract. Taking into account the very similar MW for all three eIF5A proteins in *A. thaliana*, it seems impossible to differentiate each other or their post-translational modifications from experiments based on western blot of SDS-PAGE separated proteins.

Next we tested the immunodecoration profile of a total protein extract from *A. thaliana* seedlings separated by 2D-E and compared the western blot signals with the mobility of the recombinant hypusinated eIF5A proteins fluorescently labeled. The results shown in Figure 5B allowed matching the western blot signals to the mobility of the fluorescent labeled recombinant proteins. One major signal from the western blot seems to decorate the co-migration of both eIF5A1 hypusinated protein together with eIF5A2 and eIF5A3 unmodified proteins. However, the immunoblot signal after 2D-E seems to be specific for eIF5A1, taking into account that the low amount of total protein used does not allow to reveal the presence of any eIF5A protein after silver staining, whose lowest detection threshold is in the low nanogram or subnanogram range (Chevallet et al., 2006). Although the endogenous gene expression level is higher for *eIF5A2* and *eIF5A3* genes than for *eIF5A1* (as shown in Figure 5C), the low amount of protein used for the 2D gels only allows the immunodetection of eIF5A1 protein according to the detection limit shown in Figure 5A. The most acidic signal in the western blot coincides with the non-hypusinated proteoform of eIF5A1, whereas the weakest and least acidic signal in the western blot overlaps with the mobility of both eIF5A2 and eIF5A3 hypusinated proteins. In spite of the co-migration of the non-hypusinated eIF5A1 protein with the most acidid spot detected in the western blot, we further tested whether other post-translational modification such as phosphorylation might be also involved in the altered 2D mobility. As it has been shown that eIF5A is a substrate of the protein kinase CK2 in plants (Łebska et al., 2010) we checked whether *A. thaliana* dominant negative mutants for CK2 activity (Moreno-Romero et al., 2008) would influence the 2D-E mobility of eIF5A. As shown in Figure S2, conditional inactivation of CK2 upon dexamethasone treatment leads to increased amount of the acidic eIF5A1 spot, therefore an increase in the acidic spot cannot be attributed to CK2-dependent phosphorylation activity. Moreover, these data indicate that CK2 may alter the activity of DHS enzyme *in vivo* in *A. thaliana* as it has been shown that CK2 interacts with and phosphorylates DHS in HeLa cells (Kang and Chung, 2003). These data indicate that a combination of 2D-E and western blot can be used to assess alterations in the hypusination levels of the *A. thaliana* eIF5A proteins, in particular for eIF5A1.

### ABA reduces hypusination of eIF5A1 without affecting DHS transcript levels

**Figure 6:**
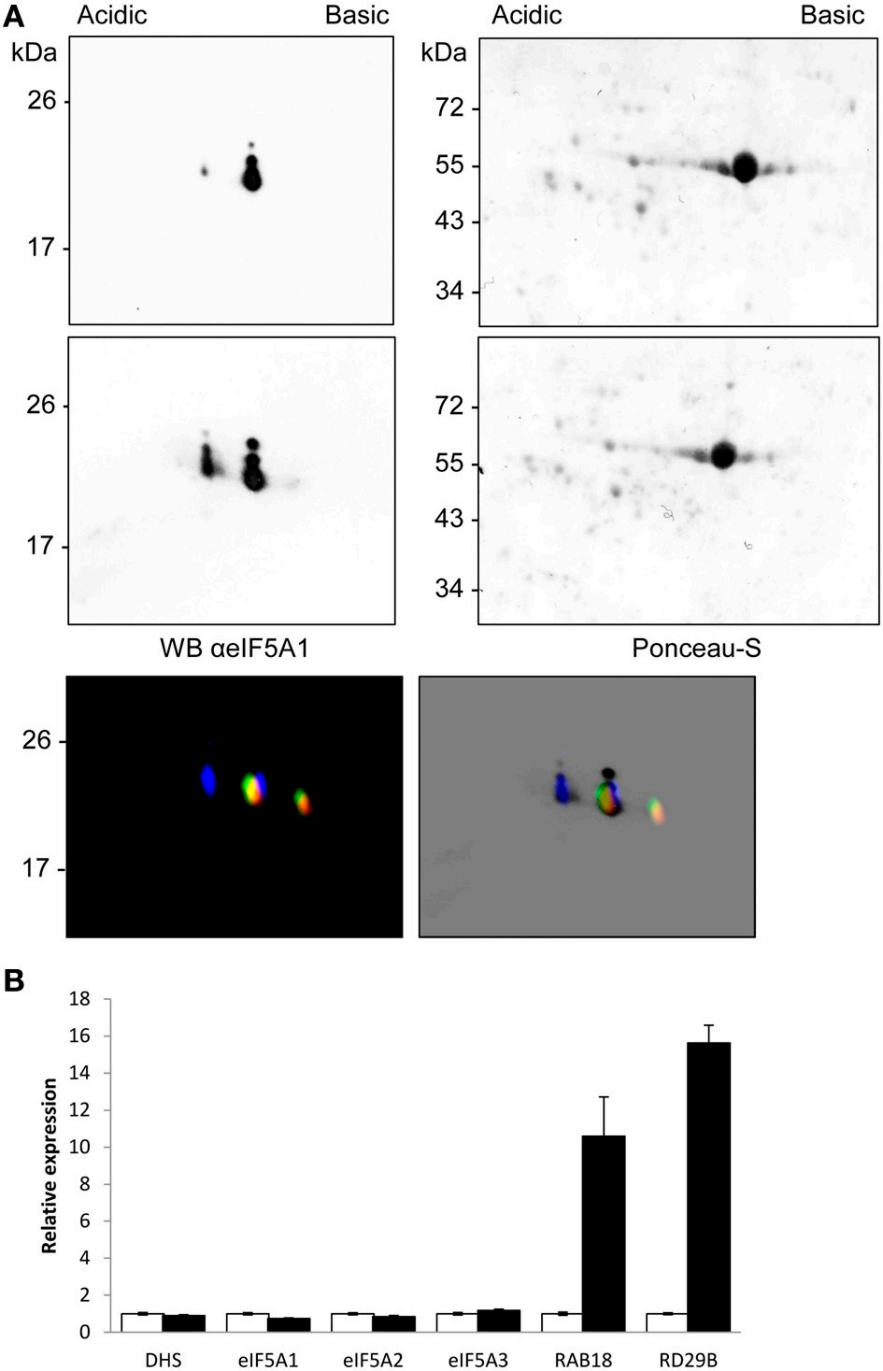
**ABA-dependent post-transcriptional inhibition of eIF5A1 hypusination**. The immunological profile of separated *Arabidopsis* eIF5A proteins was checked after ABA treatment. Control samples (upper Panel **A**) were compared to 10 μM ABA treated seedlings for 3 h (middle Panel **A**) and the mobility compared with fluorescent imaging of fluorescent labeled recombinant hypusine-modified eIF5A proteins (lower Panel **A**) as in Figure 5. The same amount of total protein from both samples was separated by 2D-E and, after Ponceau-S staining showing similar amount of Rubisco protein, the blots were used for western blot analysis with anti-eIF5A1 antibody. The western blots represent a typical result of three independent biological replicates. Panel **(B)** shows RTqPCR gene expression analysis with the same samples used for the proteomic analysis shown in **(A)**. Total RNA from control samples (white bars) and ABA-treated samples (solid black bars) were used for RTqPCR to check expression changes of eIFA genes and hypusination coding-enzyme genes together with ABA-inducible genes *RAB18* and *RD29B*; the data were referred for each gene to the non-treated control sample.

To validate the use of the proteomic tool aimed to characterize alterations in eIF5A activity, we chose plant treatments with the stress phytohormone abscisic acid (ABA), since it has recently been shown that ABA inhibits global protein translation (Guo et al., 2011). We speculated that such global alteration may also involve the activity of eIF5A. We therefore obtained total protein extracts from seedlings of the same age treated, or not, with ABA. Figure 6A shows a weak acidic spot coincident with the non-hypusinated eIF5A1 protein that increases upon ABA treatment. In the Figure 6A lower panel of the changes detected after 2DE-westernt analysis after ABA treatment were compared with the mobility of the partially hypusinated fluorescent-labeled eIF5A proteins to show a clear overlapping of the ABA-dependent increased acidic spot with the non-hypusinated eIF5A1 protein. Transcriptional analysis did not show any gene expression difference for either *eIF5A* genes or *DHS*, although ABA-inducible genes (*RAB18* and *RD29B*) were clearly induced (Figure 6B). Therefore, post-transcriptional alterations upon ABA increase may alter the activity of eIF5A by reducing its activation by hypusination and this might cause alterations in translation efficiency for the potential beneficiaries of eIF5A activity.

## Discussion

The protein translation factor eIF5A has been recently linked to the translation elongation machinery in addition to the canonical factors eEF1A and eEF2. One key feature of eIF5A relies on its post-translational activation by hypusination, as it is the only protein known that is subject to this modification. Our work aimed at the biochemical characterization of the eIF5A modification pathway in plants. Other eukaryotic systems have already been exhaustively characterized at this level (Xu and Chen, 2001; Lee et al., 2009; Patel et al., 2009; Li et al., 2010; Maier et al., 2010; Dias et al., 2013). In plants, however, much of the efforts have concentrated on genetic approaches for overexpression or antisense, but this type of studies may lead to unwished silencing and moreover a gain-of-function for eIF5A may require simultaneous coexpression of the hypusination enzymes with full activity, which has not been reported yet in plants. Therefore, it seems reasonable to think that any report considering a gain or loss-of-function for the eIF5A hypusination pathway may now include a detailed molecular characterization of its biological activity.

Until eIF5A-dependent mRNA targets are found in plants, and appropriate *in vivo* activity assays are developed, we have focused on the post-translational modification of eIF5A by hypusination that can be monitored by the use of biochemical and immunological techniques. In this work we have developed a 2D-E/western technique to evaluate differences in the relative mobility of each eIF5A protein from *A. thaliana*. We have successfully expressed recombinant eIF5A proteins as cleavable GST fusion proteins in *E. coli* both in their unmodified proteoform (Figure 1), and the hypusinated proteoform (Figures 2, 3 and Figure S1). The coexpression of the eIF5A proteins together with the hypusination enzymes facilitated the isolation of soluble proteins demonstrated to be appropriately (if not completely) modified, since the percentage of hypusinated protein was far from complete and displayed variation from one eIF5A protein to another (Figure 4). The fluorescent labeled proteins allowed us to establish a 2D-E map for all three eIF5A proteins in their modified and unmodified proteoforms (Figure 5). When combined with total protein extracts, the immunologically detected eIF5A proteins could be allocated to their hypusine-modified or non-modified proteoforms. As a proof of concept, we have shown that exogenous ABA treatments may cause inactivation of eIF5A by the increased amount of the non-hypusinated eIF5A1 protein (Figure 6). We have discarded that alterations in the mobility of the acidic spot could be due to other post-translational modifications such as phosphorylation, at least with regard to the well-established activity of the CK2 kinase that has also been shown to occur in plants (Figure S2). Other described post-translational modification that could lead to increased acidic spot for eIF5A is the acetylation of a close K to the one modified by hypusination (Ishfaq et al., 2012). Although we cannot formally exclude that such modification is detectable in *A. thaliana*, at least two additional acidic spots should be present, one highly acidic for the only acetylated proteoform and another, similar in net charge to the non-hypusinated proteoform, corresponding to the doubly modified by acetylation and hypusination in different lysines if this modification system is conserved in plants. Further experiments are required to discriminate whether both modifications are present simultaneously or under different growth conditions in *A. thaliana*. In the meantime, the biochemical characterization shown with this work can be further applied to other plant treatments or growing conditions, and are also amenable to be used for characterization of transgenic plants with genetic alterations of *eIF5A* genes.

One biological consequence of the detected ABA-dependent alteration in the hypusination of eIF5A1 is that any mRNA whose translation depends on eIF5A may suffer a translational defect under those conditions. According to the recently reported evidences in yeast that eIF5A promotes the translation of polyproline motifs (Gutierrez et al., 2013), it could be envisaged that a similar situation does happen in higher plants. A list of *A. thaliana* proteins containing at least 3 consecutive prolines yields more than 3500 which is a very large number of potential direct eIF5A targets. However, if the assumption is correct, a straightforward strategy to identify ABA-dependent eIF5A targets is to study in detail the translational profile of mRNAs encoding polyproline motifs, a true challenge for future studies in plants.

There are also a number of potential applications of the available hypusinated or non-modified recombinant eIF5A proteins from *A. thaliana*. Future studies with the hypusine-modified or non-modified recombinant eIF5A proteins may include, among others, structural analysis of the ribosome-bound proteins, *in vitro* translational studies, mRNA-binding tests, and pharmacological screenings of inhibitors of the DHS modification enzyme. As the knowledge of the eIF5A-dependent pathway increases with the identification of mRNA targets, an increase in the demand of both *in vitro* and *in vivo* approaches to characterize this pathway is expected to occur. We hope that the biochemical procedures developed and validated with this work will provide a much needed methodology to enable further studies.

### Conflict of interest statement

The authors declare that the research was conducted in the absence of any commercial or financial relationships that could be construed as a potential conflict of interest.

## Supplementary material

Figure S1 **Determination of the presence of hypusine by GCMS**. GCxGC/TOF-MS extracted ion chromatogram (m/z 375 + 174) of GST-eIF5A3 hydrolyzed protein. **(A)** Hypusinated; **(B)** non-hypusinated control; **(C)** mass spectrum of hypusine-TMS.

Figure S2 **CK2-dependent phosphorylation changes in eIF5A1 protein**. The same amount of protein extract from 10 old day *CKA3*^mut^ plants grown additionally for 3 days in the absence **(A)** or in the presence **(B)** of 10 μM dexamethasone, for CK2 conditional inactivation, were subjected to 2D-E and western blot analysis with anti-eIF5A1 antibody. Similar results were obtained in two independent technical replicates.
