# Supplementary figures and images for: Biochemical quantitation of the eIF5A hypusination in Arabidopsis thaliana uncovers ABA-dependent regulation
Source: Front Plant Sci. 2014 May 16;5:202. doi: 10.3389/fpls.2014.00202 (PMC4032925; doi:10.3389/fpls.2014.00202)

Fig.S1

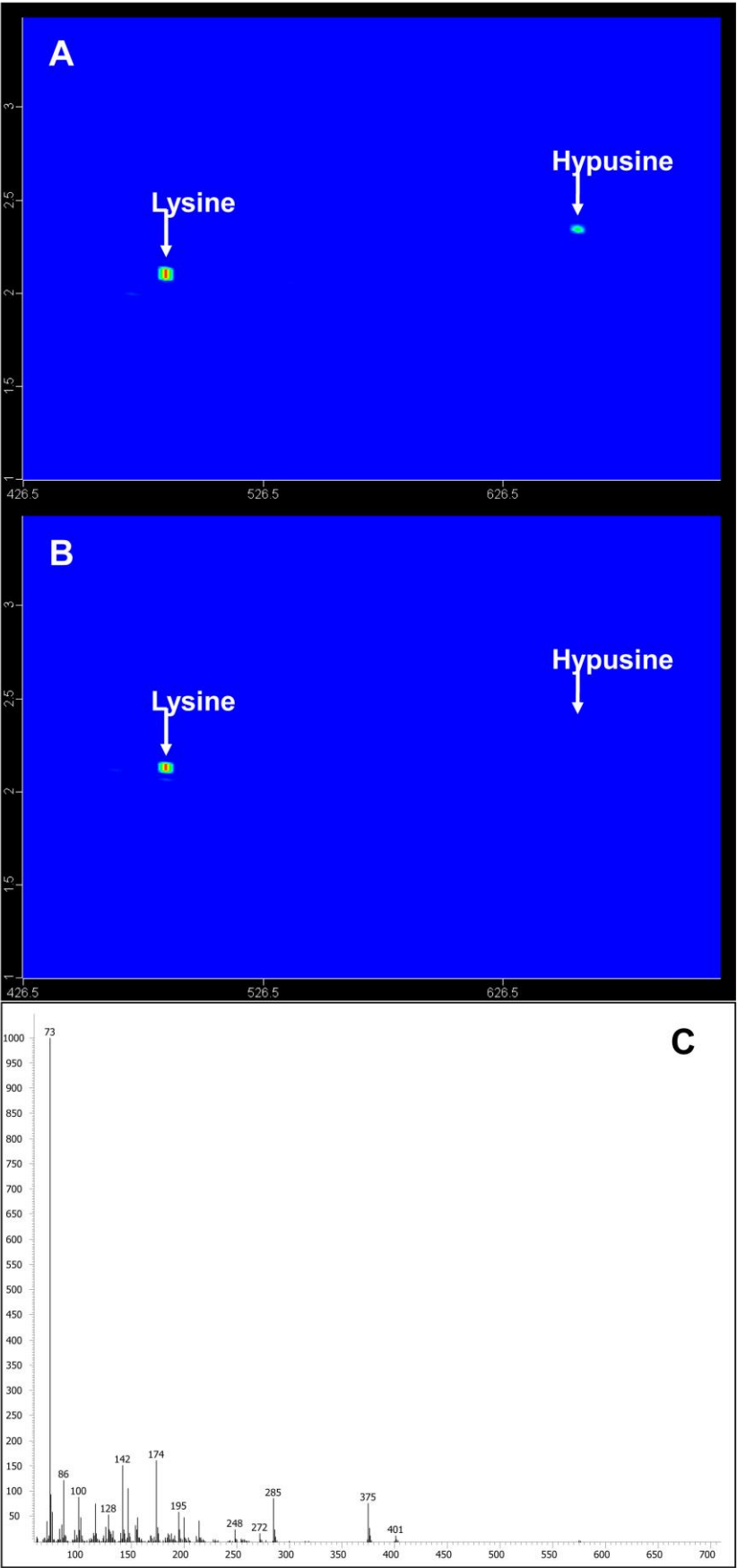

Supplement: Figure S1 — Determination of the presence of hypusine by GCMS. GCxGC/TOF-MS extracted ion chromatogram (m/z 375 + 174) of GST-eIF5A3 hydrolyzed protein. (A) Hypusinated; (B) non-hypusinated control; (C) mass spectrum of hypusine-TMS. [file DataSheet1.PDF]

Fig.S2

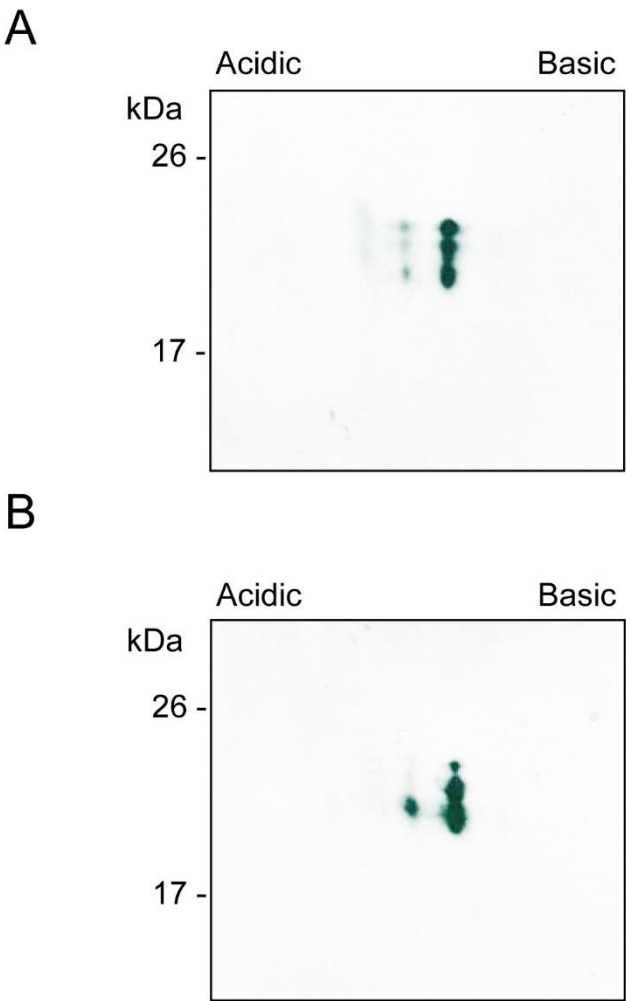

Supplement: Figure S2 — CK2-dependent phosphorylation changes in eIF5A1 protein. The same amount of protein extract from 10 old day CKA3mut plants grown additionally for 3 days in the absence (A) or in the presence (B) of 10 μM dexamethasone, for CK2 conditional inactivation, were subjected to 2D-E and western blot analysis with anti-eIF5A1 antibody. Similar results were obtained in two independent technical replicates. [file DataSheet2.PDF]
